# Cardiac arrest survivors treated with or without mild therapeutic hypothermia: performance status and quality of life assessment

**DOI:** 10.1186/s13049-014-0076-9

**Published:** 2014-12-12

**Authors:** Robert Kowalik, Ewa Szczerba, Łukasz Kołtowski, Marcin Grabowski, Karolina Chojnacka, Wojciech Golecki, Adam Hołubek, Grzegorz Opolski

**Affiliations:** 1st Chair and Department of Cardiology, Medical University of Warsaw, Banacha 1a Street, 02-097 Warsaw, Poland; Students Scientific Group of 1st Chair and Department of Cardiology, Medical University of Warsaw, Banacha 1a Street, 02-097 Warsaw, Poland

**Keywords:** Cardiac arrest, Mild therapeutic hypothermia, Outcome, Quality of life, Performance status

## Abstract

**Background:**

Hypoxic-ischaemic encephalopathy is the main determinant of clinical outcome after cardiac arrest. The study was designed to determine long-term neurological and psychological status in cardiac arrest survivors, as well as to compare neuropsychological outcomes between patients treated with mild therapeutic hypothermia (MTH) and patients who did not undergo hypothermia treatment.

**Methods:**

The article describes a single-center, retrospective, observational study on 28 post-cardiac arrest adult patients treated in the cardiac intensive care unit who qualified for MTH vs. 37 control group patients, hospitalized at the same center following cardiac arrest in the preceding years and fulfilling criteria for induced hypothermia, but who were not treated due to unavailability of the method at that time. Disability Rating Scale (DRS), Barthel Index and RAND-36 were used to assess performance status and quality of life in both study groups after hospital discharge.

**Results:**

There were no statistically significant differences in physical functioning found between groups either at the end of hospital treatment or at long-term follow-up (DRS: p = 0.11; Barthel Index: p = 0.83). In long-term follow-up, MTH patients showed higher vitality (p = 0.02) and reported fewer complaints on role limitations due to emotional problems (p = 0.04) compared to the control group. No significant differences were shown between study groups in terms of physical capacity and independent functioning.

**Conclusion:**

To conclude, in long-term follow-up, MTH patients showed higher vitality and reported fewer complaints on role limitations due to emotional problems compared to the control group. This suggest that MTH helps to preserve global brain function in cardiac arrest survivors. However, the results can be biased by a small sample size and variable observation periods.

## Background

Nearly 275,000 out-of-hospital cardiac arrests (OHCA) occur in Europe each year [1]. Sudden cardiac arrest (SCA) is an abrupt loss of heart function leading to cessation of normal circulation of the blood. The final outcome is mostly determined by the onset of hypoxic-ischaemic encephalopathy defined as irreversible, structural alterations in the central nervous system resulting from transient global hypoperfusion of the brain tissue [2,3]. It accounts for over 60% of deaths in patients hospitalized after OHCA and almost 25% of deaths in patients after in-hospital cardiac arrest following successful cardiopulmonary resuscitation (CPR) [4,5]. Mild therapeutic hypothermia (MTH) is the only currently recommended neuroprotective measure known to influence outcomes in cardiac arrest survivors. The results of a 2012 Cochrane meta-analysis show that mild therapeutic hypothermia is effective in improving neurological outcomes at the end of hospital treatment (number needed to treat [NNT] = 6) and increasing survival during hospital stays (NNT = 7) in patients following OHCA [6].

Most studies concerning induced hypothermia focus on short-term outcomes. Cerebral Performance Category Scale (CPC) is the most frequently used instrument for the evaluation of neurological status in patients following SCA. Few trials extended observation periods and integrated psychological status assessment in the study design. Quality of life and daily functioning of patients after OHCA in MTH group and in controls were compared by Bro-Jeppesen et al. using CPC, 36-item short form health survey (SF-36) and Mini Mental State Examination (MMSE) in 6 month follow-up. They described better short-term outcomes measured by CPC and no differences in long-term outcomes between the groups when comparing results from SF-36 and MMSE [7]. Wachelder et al. findings show that 74% of OHCA survivors have a low participation level in society and over 50% experience severe fatigue, 38% feelings of anxiety and/or depression and 24% a decreased quality of life [8]. Since data on the subject and the number of publications is limited, the following study was designed to determine the neurological and psychological status in cardiac arrest survivors as well as to compare neuropsychological outcomes between patients treated with MTH and patients who did not undergo MTH.

## Methods

The article describes a single-center retrospective observational study. Twenty-eight adult patients treated in the years 2011 to 2013 in the cardiac intensive care unit (CICU) following OHCA who qualified for MTH were enrolled in the study. The historical control group included consecutive 37 adult OHCA patients hospitalized at the same center between 2009 and 2011. Of note, the hypothermia programme was initiated in 2011. Inclusion in the study was not limited by an upper age limit, cardiac rhythm at baseline, or time of return of spontaneous circulation (ROSC). The type of cardiac arrhythmia leading to SCA, time of CPR initiation and time of ROSC were recorded. Both groups received the same pharmacological treatment according to post-resuscitation care guidelines.

### Hypothermia protocol

The inclusion criteria were hospitalization due to SCA, time between SCA and recruitment <4 h, age >18 years old, systolic blood pressure >80 mmHg (with or without inotropic agents), and a score in Glasgow Coma Scale (GCS) ≤8 points at the time of recruitment. Exclusion criteria included presence of diseases with short predicted life expectancy (including advanced heart failure), initial body temperature <30°C, unconsciousness prior to SCA, active bleeding, known congenital coagulation disorders, hypoglycemia, other known cause of impaired consciousness (drugs, head trauma, stroke), and pregnancy. MTH patients underwent cooling to a target temperature of 33°C by use of a patient temperature regulation system (Gaymar® Medi-therm III), adjusting temperature by supplying temperature-controlled water through a connector hose to a companion blanket. Core body temperature was monitored with an esophageal temperature probe. The temperature during the main cooling phase (36 hours) was controlled and monitored at given time intervals according to the local MTH protocol. Despite the fact that most MTH protocols use the 12–24 hour periods for main cooling phase, our protocol has extended this phase based on local experience of cardiac intensive care physicians. During the rewarming phase we aimed to achieve the temperature change rate of 0.1°C per hour.

At the time of admission, the patient’s neurological status was initially assessed using CPC and GCS. Consecutive evaluation using both scales was performed by a qualified neurologist immediately after completion of the induced hypothermia procedure and discontinuation of pharmacological sedation and analgesia. In the control group, assessment was accomplished after cessation of anaesthetic administration. Scores obtained at the end of hospital treatment were used for calculations (see Table 1 for interpretation).

**Table 1:** **Interpretation of scores in Glasgow Coma Scale and Cerebral Performance Category Scale** ***(neurological status GCS ≥13 and CPC 1–2 was considered satisfactory)***

**GCS interpretation**	**CPC interpretation**
1. *13 points and above *– mild brain damage	1. A return to normal cerebral function and normal living
2. *9*–*12 points* – moderate brain damage	2. Cerebral disability but sufficient function for independent activities of daily living
3. *8 points and below* – severe brain damage	3. Cerebral disability, limited cognition, inability to carry out independent existence
	4. Coma
	5. Brain death

CPC – Cerebral Performance Scale, GCS – Glasgow Coma Scale.

### Outcome assessment

Due to unavailability of instruments designed specifically to assess performance status and quality of life in SCA patients with hypoxic-ischaemic encephalopathy, we have employed three generally accepted tool to assess the overall health, functioning and quality of life, these included: Disability Rating Scale (DRS) [9,10], Barthel Index [11-13], and the RAND 36-Item Short Form Health Survey[14-16] and a few additional items. This assessment was preformed after hospital discharge. The authors have fulfilled the formal requirements to use the instruments, as well as obtained permission from the local ethics committee of the Medical University of Warsaw, Poland.

The DRS comprises 8 questions in four categories: arousal, awareness and responsivity, cognitive ability for self-care activities, dependence on others, and psychosocial adaptability. Each question has its own rating scale and the score is inversely proportional to the level of functioning in a given area. The total possible score ranges from 0 (no disability) to 30 (death).

The Barthel Index measures patient performance in 10 activities of daily living (feeding, grooming, dressing, bathing, toilet use, transfers from bed to chair and back, mobility on level surfaces, climbing stairs, presence or absence of urinary and faecal incontinence) and establishes the patient’s degree of independence. Scores are obtained in 5-point increments using direct observation, self-report, or responses from third parties. The total possible score ranges from 0 to 100 points, with lower scores indicating increased disability. For the purpose of the study, it was established to describe the degree of disability as mild (scores from 86 to 100 points), moderately severe (21–85 points) or severe (0–20 points).

RAND-36 includes 11 questions comprising 36 statements. The domains of role limitations due to emotional problems, energy/fatigue, emotional wellbeing, social functioning, and general health perceptions were analyzed. RAND −36 includes the same items as those in the SF-36, but the recommended scoring algorithm is somewhat different. Patients were asked to compare pre-cardiac arrest state to their current medical status. RAND allows the questionnaire to be adopted to different circumstances upon the requirements of the study.

### Data collection

**Figure 1:**
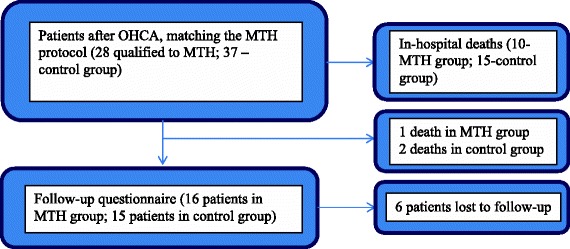
**Methods of survey data collection.**

Questionnaire answers were collected via telephone interviews in May 2013. The authors of the study made an attempt to contact all SCA survivors treated at the Department of Cardiology after 2009 (22 control group patients hospitalized in the years 2009–2010 and 17 MTH patients hospitalized between 2011 and April 2013). In June 2013, questionnaires were sent to individuals unreachable via telephone. The patients were asked to fill them out and send them back in the envelope provided before the end of July 2013. Information from 15 patients from the control group (68%) and 16 from the MTH group (94%) were received. Data concerning deaths in long-term follow-up was obtained from the national identification number database of the Polish Ministry of Interior and Administration. Six individuals were lost to follow-up (see Figure 1). The average time interval between hospital discharge and follow-up data collection was 290 (14–776) days in MTH patients and 1409 (1152–1592) days in the control group. In one case, information regarding performance and functional status was obtained from the family due to the patient’s absence.

### Statistics

Using the independent samples t-test, the MHT and control groups were compared in terms of age, duration of hospital stay (including number of days spent in the cardiac intensive care unit), time interval between cardiac arrest and the initiation of CPR, and time interval between cardiac arrest and the return of spontaneous circulation. The U Mann–Whitney test was used to compare DRS, Barthel Index, GCS and CPC, and scores for selected domains of the RAND-36 questionnaire. Correlations between respective scores were calculated using Spearman’s rank correlation coefficient. The chi-squared test was applied to compare data regarding clinical diagnosis and patient history. IBM SPSS Statistics 21.0 was used for statistical calculations.

## Results

Total mortality (CPC 5) during hospitalization and 6-month follow-up was 35.7% in the MTH and 45.9% in the control group, respectively (p = 0.49).

### Baseline characteristics

Patients representing both study groups were homogeneous in terms of demographic statistics, such as age, sex distribution, cardiac arrhythmia leading to cardiac arrest, presence of chronic conditions excluding heart failure and arterial hypertension, which were more prevalent in the MTH group (Table 2). No statistically significant differences were noted in the analyzed final discharge clinical diagnoses.

**Table 2:** **Summary of clinical characteristics of the MTH and control group patients**

	**MTH (n = 16)**	**Control group (n = 15)**	***p*** **-value (p)**
Age (years+/− SE)	55.56 ± 2.8	59.4 ± 2.9	0.358
Sex	11 males	11 males	0.779
VF/VT	16/16	13/15	0.131
Asystole	0/16	1/15	0.294
Hypertension	13/16	7/15	0.044
Diabetes	4/16	3/15	0.739
Prior myocardial infarction	2/16	3/16	0.570
Peripheral atherosclerosis	2/16	0/16	0.157
Heart failure	7/16	1/16	0.018
Coronary heart disease	4/16	2/15	0.411
CABG	1/16	0/15	0.325
AF	3/16	1/16	0.316
Renal failure	2/16	1/16	0.583
Prior stroke	1/16	0/15	0.325
Hyperthyroidism	0/16	1/15	0.294
Hypothyroidism	0/16	1/16	0.294
Smoking	9/16	6/16	0.366
COPD/asthma	1/16	1/16	0.962
Dyslipideamia	9/16	6/15	0.366
**Final diagnosis**			
STEMI	7/16	9/15	0.366
NSTEMI	2/16	3/15	0.570
Cardiac shock	2/16	4/16	0.318
Heart failure	2/16	3/16	0.570
Myocarditis	2/16	1/16	0.583

AF- atrial fibrillation, CABG – coronary artery by-pass grafting, COPD – chronic obstructive pulmonary disease, MTH - mild therapeutic hypothermia, NSTEMI – non-ST elevation myocardial infarction, STEMI – ST-elevation myocardial infarction VF - ventricular fibrillation, VT - ventricular tachycardia. p<0,05 is considered as significant.

Core body temperature at baseline averaged 35.95 ± 0.2°C. After the initial cooling phase (at the beginning of the proper cooling process), the temperature was 35 ± 0.3°C. Temperatures measured during the cooling phase at time intervals specified in the study protocol showed no statistically significant difference between individual patients and averaged 33.3 ± 0.1°C. The mean rewarming time was 871 ± 100.7 minutes until the target temperature (36.6°C) was reached.

The mean time between SCA and the initiation of CPR was 3.44 ± 2.8 minutes in the MTH group and 4.2 ± 0.6 minutes in the control group (p = 0.61). The average time between SCA and ROSC was 15.1 ± 2.9 and 11.6 ± 2.92 minutes for the MTH and the control groups, respectively (p = 0.32). The average duration of hospital stay in cardiac arrest survivors was 22.3 ± 2.81 days, including 9.6 ± 1.0 days spent in the CICU in the MTH group and 22.6 ± 4.8 days, including 9.7 ± 1.8 days in the CICU in the control group (p = 0.95 and p = 0.96, respectively).

### Outcome assessment at hospital discharge

From the group of patients from whom long-term follow-up was collected, 15 MTH patients scored ≥13 in GCS and 1 individual fell into the moderate brain injury category (GCS score 9–12) at the end of hospital treatment. In the control group, 13 patients scored ≥13 points in GCS and 2 individuals obtained between 9–12 points. None of the patients scored 3–8 points at time of hospital discharge. All study participants from whom long-term follow-up was obtained scored 1–2 points on the CPC scale at hospital discharge, independent of method of treatment. The groups did not differ in scores in GCS and CPC at baseline and at the end of hospital treatment.

### Long-term outcome assessment – data on physical functioning

The median scores for DRS and the Barthel Index in the MTH group were 0 and 100 and in the control group, 1 and 100. There were no statistically significant differences found between groups in the long-term follow-up assessed by those two scales (DRS: p = 0.11; Barthel Index: p = 0.83). Eleven out of 16 patients (68.8%) scored 0 points in DRS (no disability) in MTH group, compared to 4 out of 15 patients (26.7%) in the control group. In 10 out of 31 patients, employment prospect was impossible to assess due to retirement or pension. In the MHT group, 14 out of 16 patients (87.5%) scored over 85 points in the Barthel Index with 12 patients scoring 100 points; in the control group, 12 out of 15 patients (80%) scored 100 points in the Barthel Index (see Tables 3 and 4).

**Table 3:** **DRS results**

**DRS score**	**Level of disability**	**Control group (n)**	**MTH (n)**
0	None	5	11
1	Mild	3	1
2-3	Partial	4	2
4-6	Moderate	0	0
7-11	Moderately severe	3	1
12-16	Severe	0	1
17-21	Extremely severe	0	0
22-24	Vegetative state	0	0
25-29	Extreme vegetative state	0	0

DRS – Disability Rating Scale, MTH – mild therapeutic hypothermia, n-number of patients.

**Table 4:** **Barthel index results**

**Barthel index score**	**Degree of impairment**	**Control group (n)**	**MTH (n)**
86-100	Mild	12	14
21-85	Moderately severe	3	2
0-20	Severe	0	0

MTH – mild therapeutic hypothermia, n-number of patients.

### Long-term outcome assessment – data on psychosocial functioning and quality of life

In long-term follow-up, MTH patients showed higher vitality (p = 0.02) and reported fewer complaints on role limitations due to emotional problems (p = 0.04) compared to the control group (Table 5). No differences were noted in comparison in domains of emotional wellbeing and social functioning. No statistically significant differences were noted in self-assessed health change between present and pre-cardiac arrest state (p = 0.29): 4 out of 16 MTH patients vs. 2 out of 15 patients from the control group described their health status as considerably or slightly better compared to pre-cardiac arrest state. Eight out of 16 MTH patients vs. 5 out of 15 control group patients described their status as comparable, and 4 out of 16 vs. 8 out of 15 patients described it as slightly or considerably worse.

**Table 5:** **RAND-36 questionnaire results (the result expressed as rank average is directly proportional to the number of negative assessments of a given domain and inversely proportional to the level of patient functioning in the respective area)**

**RAND-36 domains**	**Control group (rank average)**	**MTH (rank average)**	**p (U-test)**
Role limitations due to emotional problems	18.83	12.17	0.037
Energy/fatigue	16.92	10.08	0.022
Emotional wellbeing	15.07	12.85	0.488
Social functioning	17.59	14.30	0.318
General health	17.71	12.47	0.102

MTH – mild therapeutic hypothermia, p<0,05 is considered as significant.

Patients from the control group more frequently reported problems with everyday life functioning (6/15 patients vs. 3/14 patients, p = 0.04; two patients from the MTH group did not answer this item). In the whole study group, these problems included physical disability, impaired memory, problems with emotion control and logical reasoning, sexual dysfunction, low self-esteem, and fear of another incident of arrhythmia or death. The two groups did not vary in terms of post-cardiac arrest rehabilitation services use, the number of rehospitalizations or use of nursing care. Only 9 patients, including 4 MTH (29%) and 5 control group (33%) individuals, used rehabilitation services. Two patients representing both study groups received nursing care. Seven out of 28 individuals (4 MTH and 3 control group patients) were rehospitalized due to complications following cardiac arrest.

A positive correlation was observed between DRS score and vitality (r = 0.405; p = 0.04), as well as between DRS score and social functioning (r = 0.391; p = 0.03). Additionally, a trend was shown between general health perceptions and DRS score (r = 0.351; p = 0.06). A negative correlation was observed between Barthel Index scores and the level of social functioning (r = −0.405; p = 0.02).

## Discussion

The primary goal of the study was to obtain information on the degree of independence in everyday life functioning of cardiac arrest survivors and their self-assessment of health status before and after the incident. No significant differences were shown between groups in terms of physical capacity and independent functioning as measured by DRS and Barthel Index. This appears to be associated with favorable neurological outcomes in both study groups at the time of hospital discharge: all study participants scored 1 or 2 points in the CPC scale, while 15/16 MTH patients and 13/15 control group patients scored ≥13 points in GCS. Patients who died after hospital discharge had a worse neurological status at the end of hospital treatment. In terms of psychological functioning, individuals undergoing MTH were found to have higher levels of vitality and fewer limitations due to emotional problems compared to the control group. This may indicate that MTH after SCA helps to preserve global brain function and results in fewer impairments of functions which require global brain involvement.

These observations are consistent with the results obtained by Elliott et al. [17], who analyzed data from 70 trials investigating quality of life after SCA in diverse populations. These trials varied with regard to quality of life assessment methods and follow-up periods that ranged from 0 (evaluation at time of hospital discharge) to 15 years. Forty-six trials have demonstrated good quality of life in SCA survivors following hospital treatment, whereas 7 proved it to be poor. In the remaining 17 studies, quality of life was described as neutral due to social isolation, cognitive impairment, depression, or anxiety. A secondary conclusion from this paper concerned the variety of methods applied in the analyzed studies that ranged from recognized assessment tools to self-invented quality-of-life evaluation surveys, as well as structured or unstructured patient or family interviews. Adopted tools differed in terms of specificity, ranging from general scales, such as CPC, to neurocognitive test batteries designed to provide complex neuropsychological assessments [17]. As opposed to data cited above, Reinhard et al. [18] demonstrated worse quality of life in cardiac arrest survivors compared to the general population as assessed by RAND-36. In long-term follow-up (16–62 months after the incident), SCA survivors showed significantly worse performance in terms of general health perceptions, physical capacity, social functioning, and role limitations due to emotional problems compared to general population [18]. The studies described above analyzed results of OHCA survivors treated without MTH. The number of publications regarding therapeutic hypothermia treatment remains even smaller. One of the studies by Storm et al. [19] focused on patients with ventricular fibrillation SCA and assessed neurological outcome in the MTH vs. control (normothermic) group. The results demonstrated that patients undergoing MTH scored higher on the CPC scale at time of hospital discharge compared to the control group. MTH patients showed better survival rates in a 2-year follow-up compared to individuals treated with other methods. Additionally, patient age and the adoption of therapeutic hypothermia were proven to be the only factors influencing 2-year survival [19]. Bro-Jeppesen et al. [7] analyzed quality of life and daily functioning of patients after OHCA in MTH group and in controls using CPC, SF-36 and Mini Mental State Examination (MMSE) in 6 month follow-up. In long-term follow-up they did not observe differences between the groups when comparing results in SF-36 and MMSE. However in this study detailed dimensions of SF-36 were not analyzed, and the authors observed a trend toward lower scores in both sub-scores of SF-36 (role functioning and role emotion) in patients treated without MTH which is similar to our results. In study by Bro-Jeppesen et al. only patients with initial VF/VT rhythm were interviewed in the long-term follow up [7].

In the presented study, positive assessment of health status was associated with better physical capacity as measured by DRS. Patients who scored higher on scales measuring performance showed better social functioning. No statistically significant differences in general health status were observed between groups when patients were comparing the follow-up period with prior to the cardiac arrest status. However, as many as 25% of the MTH patients described their health status as considerably or slightly better compared to pre-cardiac arrest state vs. 13% in the control group. Almost 50% of patients from the MTH group vs. 33% of the control group patients assessed their health as comparable, whereas 25% vs. 53% described it as slightly or considerably worse. These results are comparable with the ones reported by Wachelder et al. [8] who describe long-term outcomes of OHCA survivors treated in years 2001–2006. Quality of life assessed by SF-36 was decreased in 24% of patients in mean time follow-up of three years. On contrary to our findings Wachelder et al. describe more cognitive problems in patients treated with MTH. In discussion however they underline that this may be to selection bias which we avoided in our study by including patients with similar GCS score upon admission [8]. Some of the reported complaints of patients after OHCA can be suggestive of clinically overt depression or post-traumatic stress disorder, both of which tend to occur following cardiac arrest [20].

As was previously mentioned, due to the limited number of publications concerning the physical and psychosocial status of cardiac arrest survivors, independent of the method of treatment, it proves difficult to relate our study results to existing literature. Adequate measurement tools dedicated specifically to this group of patients are also lacking. The scope and degree of central nervous system functional impairment resulting from post-cardiac arrest encephalopathy is difficult to predict, as it depends on a number of factors, including duration of hypoperfusion and hypoxia, and individual capacity for restoration of neuropsychological functions. Restriction of blood flow appears to cause brain injury in a different mechanism of action than the absence of oxygen supply. Ischeamia caused by brain hypoperfusion leads to infarctions at the border zones between major cerebral arterial territories, whereas anoxia results in a more diffuse neuronal injury, affecting various brain regions, e.g. hippocampus or cerebral cortex. Anoxic injury can further be aggravated by a developing brain oedema and damage caused by the restoration of blood flow, which can be prevented by the induction of an almost routinely used mild therapeutic hypothermia [21,22].

ILCOR recommendations state that unconscious adult patients with spontaneous circulation after OHCA should be cooled to 32°C to 34°C for 12 to 24 hours [23]. In our study the duration of MTH was 36 hours. This longer time of cooling is recommended by Polish Registry of Therapeutic Hipothermia Guidelines [24]. In 2010 a survey evaluating level of therapeutic hypothermia implementation in intensive care units in Poland was carried out. According to this registry up to 26% ICUs continued therapeutic hypothermia for longer that 24 h in patients after cardiac arrest [25]. In comparison in United Kingdom 13% of ICUs maintained cooling phase for >24 hours after cardiac arrest [26]. There are several data that point to potentially beneficial impact of longer-lasting MTH. In rats after induced cardiac arrest it has been shown that therapeutic hypothermia lasting 48 h compared to 24 h correlated with greater surviving neuron count [27]. A small sample study suggests that MTH lasting 72 h after cardiac arrest possibly blunts the inflammatory response after rewarming and may lower secondary brain injury [28]. In infants with hypoxic-ischemic encephalopathy, hypothermia of 33- 34°C lasting for 72 h is considered safe, improves survival rate and neurodevelopmental outcome [29]. In traumatic types of brain injury following cerebral contusions and elevated intracranial long-term hypothermia, up to 5 days significantly improves outcomes in patients without causing significant complications [30]. On the other hand Shinozaki et al. [31] found out that the optimal duration of well-controlled hypothermia (33°C ± 1°C) is over 18 hours and independently correlates with favorable neurological outcome at 6 month follow-up in patients after OHCA.

Key limitations concerning assessment of physical functioning, performance, and quality of life in cardiac arrest survivors in long-term follow-up in the presented study include a small study population and variable observation periods. This trial should be regarded as pilot study and large, randomized trials using relevant existing instruments or evaluation tools developed specifically for patients following cardiac arrest should be performed to provide broader information. Our results might be partly influenced by the fact that due to organizational reasons only 9 out of 25 patients received a post-hospital rehabilitation, a treatment that has been proved to significantly improve long-term outcomes. Another limitation of our trial is the fact that the collection of responses to the questionnaires has been performed even as long as fours years after the index incidence. This may be a confounding factor more pronounced for the historical cohort that was hospitalized between 2009 and 2011. Presented results may be also altered by varied time of follow-up period ranging from 14 to 776 days in the MTH group. The questionnaires themselves have also several limitations. In small population samples the results of DRS, Barthel Index and RAND-36 can be difficult to interpret due to floor and ceiling effect. In our group we observed that high percentage of patients achieved 100 points in Barthel Index and 0 points in DRS pointing to good functioning in the examined aspects of life. Those questionnaires do not allow to diagnose subtle deficits in physical, independent functioning.

## Conclusion

In conclusion, as compared to historical cohort of patients after OHCA, patients treated with MTH report higher vitality and less emotional constrains. No significant differences were observed between study groups in terms of physical capacity and independent functioning. A dedicated health related quality of life tool is needed to precisely and adequately assess patients treated with MTH. Further randomized, controlled trials are needed to confirm the above results in the era of dedicated cooling devices.

## Abbreviations

CICU: cardiological intensive care unit
CPC: Cerebral Performance Category Scale
CPR: cardiopulmonary resuscitation
DRS: Disability Rating Scale
GCS: Glasgow Coma Scale
MTH: mild therapeutic hypothermia
NNT: number needed to treat
OHCA: out-of-hospital cardiac arrests
RAND-36: RAND 36-Item Short Form Health Survey
ROSC: return of spontaneous circulation
SCA: sudden cardiac arrest
